# Association between transforming growth factor β1 polymorphisms and atrial fibrillation in essential hypertensive subjects

**DOI:** 10.1186/1423-0127-17-23

**Published:** 2010-03-31

**Authors:** Yongzheng Wang, Xuwei Hou, Yuliang Li

**Affiliations:** 1 Department of Interventional Radiology, The Second Hospital of Shandong University, Shangdong, PR China; 2 Department of Cardiology, The First People Hospital of Hangzhou, Hangzhou, PR China

## Abstract

**Background:**

The association of TGF β1 polymorphisms and atrial fibrillation (AF) in essential hypertensive (EH) subjects remains unknown. Methods EH subjects with AF (EH+AF+) and sinus rhythm (EH+AF-) were enrolled. The polymorphisms of +869 T → C at codon 10 and + 915 G → C at codon 25, were genotyped. The clinical characteristics including serum TGF β1 levels were detected.

**Results:**

The GG genotypes of TGF β1 +915 G → C at codon 25 were more prevalent in subjects from EH+AF+ group than those from EH+AF- group (P = 0.009). The subjects with GG genotype from EH+AF+ group had the highest mean serum TGF β1 level, which was significantly higher than that of GG genotype subjects from EH+AF- group (3.18 ± 0.24 ng/dl vs.2.29 ± 0.14 ng/dl, P < 0.05). Multiple analyses revealed that the TGF β1 GG genotype of +915 G → C at codon 25 presented a 3.09 times higher risk in developing AF in the multivariate model after adjusting for age and gender.

**Conclusion:**

The polymorphisms of TGF β1 +915 G → C at codon 25 were associated with occurrence of AF and serum TGF β1 level in EH subjects.

## Background

Atrial fibrillation (AF) is a common and clinically important arrhythmia in practice, which represents a major public health problem. AF induces hemodynamic impairment and thromboembolic events, resulting in significant morbidity, mortality, and cost [1,2].

A number of factors, e.g. age, coronary artery disease, myocardial infarction, heart failure, valvular heart disease, contribute to the occurrence and development of AF [3,4]. In addition, population based studies revealed that hypertension is an independent risk factor for onset of AF [5]. The risk of developing AF in hypertensives was 1.9 times higher than normtensives in the Framingham Heart Study [6].

The precise mechanism of AF remains largely unknown. Compelling evidence showed that the atrial fibrosis is essential for the onset and maintenance of AF [7]. Atrial fibrosis causes conduction abnormalities which results in an increase in AF vulnerability. Increased atrial fibrosis was observed in the biopsy and autopsy specimens from patients with AF [7-15].

Transforming growth factor β1, (TGF β1) is a cytokine that modulates the tissue fibrosis. Previous study showed that over-expression of TGF β1 selectively induced atrial interstitial fibrosis, contributing to AF vulnerability [16,17]. Inhibition of TGF β1 expression by certain drug decreased the atrial fibrosis and AF vulnerability[18]. These studies suggest that TGF β1 play an essential role in inducing AF.

The expression of TGF β1 is under gene control. Several functional polymorphisms in the TGF β1 gene had been determined previously. Some of these functional polymorphisms, e.g. (+869 T → C at codon 10 and +915 G → C at codon 25) are reported to be associated with cardiovascular disorders, including myocardial infarction, artery stiffness and LVH in hypertensives [19-25].

To date, the association between TGF β1 gene polymorphism and the occurrence of AF in hypertensive subjects remains unknown. We hypothesized that the TGF β1 polymorphisms genetically determined the predisposition to AF in hypertensives. In current study, we recruited newly diagnosed essential hypertensives with and without AF to testify this hypothesis.

## Methods

### Subject Enrollment

Newly diagnosed essential hypertensive subjects were enrolled in this study. The subjects with documented AF were assigned into the EH+AF+ group and those with sinus rhythm were assigned into the EH+AF- group. To avoid any possible influence of certain anti-hypertensive drugs on the onset of AF, all subjects received no treatment when they were enrolled. Hypertension was defined as systolic blood pressure (SBP)> = 140 mm Hg, or diastolic blood pressure (DBP)> = 90 mm Hg in supine position, after 20 min of rest on 2 separate days. AF was determined by 12-lead electrocardiography (ECG) and/or 24-h Holter monitoring. Prior or current documented permanent or paroxysmal AF was considered as AF subjects. Clinical characteristics such as age, sex, body mass index (BMI), and smoking status were collected. Patients with secondary hypertension, coronary heart disease, diabetes myocardial infarction and/or other significant heart problems, such as severe valvular heart disease, dilated phase HCM, congenital heart disease, having other types of arrhythmia, was excluded. Informed consent was obtained from each subject and the Institutional Ethninc Board of the university approved the study.

### Plasma measurements

Blood was collected at morning from resting and fasting subjects. Lipid profiles (total cholesterol, TC and triglycerides, TG) were determined by enzymatic-colorimetric methods according to manufacturer instructions on a Beckman spectrophotometer (Beckman, USA). LDL-C was calculated by the Friedewald's formula. The serum C reaction protein (CRP) concentration was measured by high sensitivity enzyme immunoassay (Dade-Behring, Marburg Germany) for the quantitative determination.

### Serum TGF β1 detection

The serum TGF β1 was measured using the BDA19 capture ELISA as described previously[26]. The intra-assay coefficient of variation of the assay used is 6.8% and the sensitivity (defined as 2 SD above the mean of 16 blank determinations) was ~0.1 ng/ml.

### Genotyping

DNA was isolated from the whole blood according to standard procedures. Genotyping of the TGF-1 polymorphisms of the +869 T → C at codon 10 and +915 G → C at codon 25 was performed. Briefly, 20 μL of genomic DNA solution was added to D-mix, which contains the dNTPs and reaction buffer, for the cytokine genotyping. Taq polymerase (1.1 μL; Gibco BRL, USA) was then added to the D-mix, vortexed for 15 seconds, and 10 μL of the D-mix mixture transferred to a 96-well microtiter genotyping tray with dried primers in each reaction well. A Perkin-Elmer 9600 thermocycler was used to amplify the promoter regions by PCR. Samples were subjected to 10 cycles at 96°C for 10 seconds, and 63°C for 60 seconds, followed by 20 cycles at 96°C for 10 seconds, annealing temperature of 59°C for 50 seconds, and 72°C for 30 seconds. After the PCR process, the amplified DNA fragments were separated by agarose gel electrophoresis and visualized by staining with ethidium bromide and exposure to ultraviolet light in an UV transilluminator.

### Statistical analysis

All data were analyzed by SPSS (version 13.0) software. The clinical characterstics between EH+AF+ and EH+AF- were compared by *t *test. The Serum TGF β1 levels according to the genotype distributions were performed by the ANOVA test and pos hoc analysis. The genotype distributions and allele frequencies of TGF β1 in two groups were evaluated by χ^2^-test. Logistic regression analysis was performed to assess the odd ratio (OR) for AF in EH subjects. P value ≤ 0.05 was considered statistically significant.

## Results

The clinical and biochemical data of all subjects were listed in Table 1. There were no significant differences in age, sex, height, weight, BMI, SBP, DBP, serum TG, TC, HDL-C, and LDL-C between EH+AF- and EH+AF- groups. The mean serum CRP level was markedly higher in the EH+AF+ group than in the EH+AF- group. Smokers were more prevalent in EH+AF+ group than in EH+AF- group. The mean serum TGF β1 levels did not show significant difference between two groups.

**Table 1:** Clinical and biochemical characteristics of all subjects

	AF+	AF-	P
Age (years)	45.6 ± 6.7	46.1 ± 4.9	NS

Height (cm)	175.4 ± 8.5	175.2 ± 6.4	NS

Weight (kg)	58.6 ± 9.2	59.1 ± 5.8	NS

Smoker (%)	66.4	47.8	0.02

SBP (mmHg)	155.6 ± 11.4	153.9 ± 9.9	NS

DBP (mmHg)	89.6 ± 6.8	90.6 ± 7.5	NS

BMI	25.6 ± 1.6	25.9 ± 2.1	NS

TG (mg/dl)	122 ± 13.7	124 ± 9.6	NS

TC (mg/dl)	196.6 ± 14.8	200.5 ± 16.3	NS

HDL-C (mg/dl)	49.8 ± 5.8	51.5 ± 8.2	NS

LDL-C (mg/dl)	113.7 ± 10.8	111.6 ± 8.1	NS

CRP (mg/dl)	2.116 ± 0.08	1.081 ± 0.06	<0.001

TGF β1 (ng/ml)	2.23 ± 0.12	2.22 ± 0.16	NS

Table 2. showed the genotype distributions and allele frequencies of TGF β1 in two groups. All the allele frequencies fit in with the Hardy-Weinberg equilibrium law. For the polymorphisms of + 915 G → C at codon 25, the GG genotype was more prevalent in the EH+AF+ subjects than in the EH+AF- subjects (P = 0.009). For the polymorphisms of +869 T → C at codon 10, no significant difference were noted between the two groups (P = 0.075).

**Table 2:** Distributions of genotype distribution and allele frequenies of TGF β1

	EH+Af+ (n = 240)	EH+Af- (n = 300)	X^2^	P
***Condon 25 G/C***				

GG	106	94	9.54	0.009

GC	74	110		

CC	60	96		

***Condon 10 T/C***				

TT	87	108	0.52	0.975

TC	79	97		

CC	74	95		

**Figure 1:**
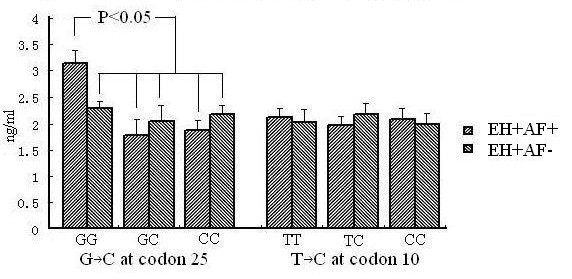
**Serum TGFβ1 level according to the genotype profile**.

Figure 1. showed the serum TGF β1 levels according to the genotype profiles. Although Table 1. showed no significant difference Iin the overall mean TGF β1 levels between EH+AF+ and EH+AF- groups (2.23 ± 0.12 vs.2.22 ± 0.16, NS), we observed that the subjects with GG genotype from EH+AF+ group had the highest mean serum TGF β1 level, which was significantly higher than that of GG genotype subjects from EH+AF- group (3.18 ± 0.24 vs. 2.29 ± 0.14, ng/dl, P < 0.05). When it was compared to the GC and CC genotypes from both EH+AF+ and EH+AF- groups, statistically differences were noted as well (all P < 0.05). For the genotypes from the +869 T → C at codon 10, the mean TGF β1 levels were similar among subjects with different genotypes in both EH+AF+ and EH+AF- groups.

Table 3. showed the odd ratio (OR) for AF in EH subjects. As determined by the logistic regression analysis, the TGF β1 GG genotype presented a 3.09 times higher risk in developing AF in the multivariate model after adjusting for age and gender. As shown in Table 3., the other risk factors for AF subjects included age, CRP level and smoke.

**Table 3:** OR to AF determined by logistic regressiona analysis

Variable	Odd Ratio (95% CI)	P
TGF β1 GG genotype	3.09 (2.11-6.59)	<0.01

Age (years)	1.45 (1.11-2.32)	0.04

CRP (mg/dl)	1.81 (1.15-3.49)	0.03

Smoker (%)	2.47 (1.98-4.66)	0.01

## Discussion

The present study assessed the association between the single nucleotide polymorphisms at the TGF β1 locus and AF in subjects with essential hypertension. We found that the GG genotype of TGF β1 +915 G->C at codon 25 was more prevalent in the individuals with AF than those without. Multiple analyses revealed that the GG genotype carriers presented an odd ratio of 3.09 for developing AF. The +869 T->C at codon 10 showed no positive relation with AF. As far as we know, this is the first study regarding the association between the TGF β1 polymorphisms and AF in hypertensives.

TGF β1 is a cytokine that regulates the synthesis of extracellular matrix components such as collagen, fibronectin, and proteoglycan. The role of TGF β1 in cardiac fibrosis and AF had been studied. Over-expression of TGF β1 selectively induced atrial fibrosis, leading to increased conduction heterogeneity and AF vulnerability without affecting the cellular electrophysiology[16]. Inhibition of TGF β1 expression by pirfenidone (PFD) significantly reduced the atrial fibrosis, as a result, reduced conduction abnormalities and AF vulnerability were observed[18]. These studies suggest that the TGF β1 attribute to development of AF via triggering atrial fibrosis. Higher levels of serum or plasma TGF β1 have been observed in subjects with hypertension, in association with cardiac and renal complications [27-31]. The TGF β1 condon 25 polymorphisms are located in the signal peptide sequence, which regulate the export of synthesized TGF β1 protein across membranes of the endoplasmic reticulum and the activation of protein. Previous studies showed that the TGF β1 levels of subjects with GG genotype were markedly higher than those with GC and CC genotypes in heart and lung transplant patients[32,33]. In consistent with these studies, we found that the subjects with codon 25 G/G genotype had higher TGF β1 plasma level in subjects from EH+AF+groups than those with the same genotype from EH+AF- groups, although no significant difference of overall mean TGF β1 levels between EH+AF+ and EH+AF- groups was observed.

In the ECTIM Study, Rao et al. reported the G/C genotype at codon 25 provided a 2.3-fold greater risk for the presence of vascular disease in hemodialysis patients[22]. Xu and his colleagues reported genetic role of TGF β1 Arg25Pro polymorphisms (GC genotype in present study) in the occurrence of left ventricle hypertrophy in EH subjects [25]. Our data showed that the GG genotype, rather than GC genotype, was related to AF incidence in EH subjects. This inconsistency may be explained in part by the difference in study protocol and relatively small scale samples in these studies.

All EH subjects in our study were newly diagnosed and none of them received anti-hypertensive treatment at enrollment. This is important because some antihypertensive agents, e.g. β blockers and angiotension converting enzyme inhibitors and Angiotensin II receptor blockers may inhibit the onset and maintenance of AF.

Taken together, in present study we found the GG genotype of TGF β1 +915 G->C at codon 25 was associated with occurrence of AF in EH subjects. This finding may help to evaluate the risk of developing AF in EH patients for a reinforced prevention.

## Competing interests

The authors declare that they have no competing interests.

## Authors' contributions

YL participated in the design of the study. YW, XW and ZL conducted the serum TGF β1 detection and genotyping, YW wrote the manuscript. XH and XS performed the statistical analysis. All authors read and approved the final manuscript.
